# Characterization of 19 new microsatellite loci for the Omani barb *Garra barreimiae* from 454 sequences

**DOI:** 10.1186/1756-0500-7-522

**Published:** 2014-08-12

**Authors:** Sandra Kirchner, Thomas Weinmaier, Thomas Rattei, Helmut Sattmann, Luise Kruckenhauser

**Affiliations:** Natural History Museum Vienna, Central Research Laboratories, Burgring 7, 1010 Vienna, Austria; Division of Computational Systems Biology, Department of Microbiology and Ecosystem Science, University of Vienna, Althanstraße 14, 1090 Vienna, Austria; Third Zoological Department, Natural History Museum Vienna Austria, Burgring 7, 1010 Vienna, Austria; Natural History Museum Vienna, Central Research Laboratories, Burgring 7, 1010 Vienna, Austria

**Keywords:** *Garra barreimiae*, *Garra rufa*, Oman, microsatellites, 454 sequences

## Abstract

**Background:**

*Garra barreimiae* is a cyprinid fish from the southeastern Arabian Peninsula, which inhabits regularly desiccating wadis and survives in isolated ponds or underground. In 1984 a cave-dwelling population was found in the Al Hoota cave system and previous genetic analyses revealed some differentiation with limited gene flow between the surface populations and the cave population. Since no suitable markers are available for evaluation of gene flow between the cave population and the adjacent surface populations, we focused on designing and establishing novel microsatellite markers from next generation sequencing data.

**Findings:**

19 microsatellite markers containing di- and tetranucleotide simple sequence repeats were developed from 454 sequences. Forty-four individuals from two surface populations (Wadi Al Falahi and Misfat Al Abriyeen) of *G. barreimiae* (sampling permission number 13/2012, export permission number 29/2012) were used for analyses and characterization of the loci. On average, the number of alleles per locus is 7.6 (range: 2–20). Two markers displayed indication of linkage disequilibrium in both populations (DL6X, 9XNC). Significant deviation from Hardy-Weinberg equilibrium was observed at four loci in the Misfat Al Abriyeen population (2PUM, 88CM, 1EHE, 3Z7M) and at two loci in the Wadi Al Falahi population (QLIM, 3 N43). Three of the microsatellite loci were significant for null alleles in one of the two populations (Misfat Al Abriyeen: CJHG; Wadi Al Falahi: PH8A, 3ROZ). Expected and observed heterozygosities ranged from 0 to 95.0% respectively from 0 to 95.8% (Wadi Al Falahi) and from 0 to 89.1% respectively from 0 to 95.0% (Misfat Al Abriyeen). Fourteen of these markers were successfully cross-amplified in *G. rufa*.

**Conclusion:**

This 19 microsatellite loci provide a useful tool to understand the structure and genetic differences of populations. Moreover, these markers will help to evaluate species delimitation in *G. barreimiae* and potentially even in related species.

## Findings

The cyprinid *Garra barreimiae* Fowler & Steinitz (1956) is endemic to the southeastern Arabian Peninsula, where it is common in ponds and rivulets of the Hajar mountains in the northern Oman and the United Arab Emirates [1] [2]. Most of these wadis dry out during summer and are only occasionally flooded, so the fish have to survive in isolated ponds or underground. In 1980 a hypogean troglomorphic population of this species was found in the Al Hoota cave system near Al Hamra [3]. All adult individuals in this population are missing externally visible eyes, and the optic lobes are reduced. In addition, the squamation is weak and they lack pigmentation [3]. Until now the Al Hoota cave population is the only known troglomorphic form of *G. barreimiae*. Previous genetic analyses on the basis of the mitochondrial control region revealed a differentiation between cave and surface populations and possible gene flow as indicated by shared haplotypes [4]. The separation is not complete, and there seems to be limited gene flow from the cave to the adjacent surface populations. The small genetic differentiation between the two forms indicates that the troglomorphic cave population is of quite recent origin [4]. According to current taxonomy, the species comprises three subspecies: *Garra barreimiae barreimiae*, which can be found throughout the northern Oman, *G. b. gallagheri* Krupp [5] found only at one location in northern Oman and *G. b. shawkahensis* Banister & Clarke [1], found north of the United Arab Emirates [5]. Investigations of Kruckenhauser et al. [4] revealed that the first two subspecies are genetically distinct based on mtDNA analyses. Moreover, they found an additional clearly separated clade on the northeastern slope of the Hajar Mountains, suggesting that *Garra barreimiae* may be comprised of more than one species. In the present study, we analysed individuals of *G. barreimiae* from two surface populations: Misfat Al Abriyeen is a locality near the mountain village Misfat, north of Bahla. The fish were collected in the Falaj drainage system, which channels the water through the villages’ gardens. About 15 km southwards from Misfat the small water body Wadi Al Falahi, a natural drain from the surrounding mountains, is located. Both localities hold numerous individuals of *G. barreimiae*. The individuals of *G. rufa*, which were used to test whether cross-species amplification works in another species of the genus, were commercially acquired.

To estimate gene flow between the different morphs and populations for this – in terms of evolutionary biology – highly interesting fish species specific genetic markers are needed. The method of choice for this purpose are microsatellite markers, which however, were up to now not available for *G. barreimiae* [6]. Next generation sequencing allows the isolation of a high number of microsatellite loci at moderate costs [7] and multiplexing of loci enables high through-put genotyping. Here, we describe the development of 19 microsatellite markers for *G. barreimiae* using the new technological advantages.

For DNA extraction, we used tissue from a finclip taken from one specimen of the cave population. We followed the DNA-extraction protocol (GEN-IAL First-DNA All-tissue DNA-Kit, GEN-IAL GmbH, Germany) and isolated genomic DNA. Approximate 500 ng DNA was used for 1/20 of a plate for 454 sequencing on a Roche/454 GSFLX sequencer at the Genomics Core Facility University of Leicester, UK. Sequencing generated a total of 36126 reads. The sequence reads were assembled into 193 contigs with size ranging from 500 – 3547 bp by using Newbler (454 Life Sciences, Roche, Switzerland). Of the remaining 35503 non-assembled reads, we also considered 29418 high quality reads (3’ trimmed until quality 20; average quality 20; minimal length 100 bp) with sizes between 100 and 731 bp.

We designed primers with the program QDD version 2.1 [8], which incorporates the computer language Perl and the programs ClustalW2 [9], Primer3 [10] and BLAST + [11]. Criteria used to select microsatellite loci included: appropriate product size (between 75 and 320 bp) and a minimum repeat motif of five subunits consisting of two to six nucleotides. For the primer design, conditions were set for a 20 bp optimum length (18–27 bp) and optimum temperature of 60.6°C, allowing a 12°C temperature difference between the two primers of a primer pair (52-63°C). Optimum GC-content was set on 50% (20–80). After these selections 919 contigs and single reads with at least one microsatellite and featuring suitable primers fulfilled the requirements. After selection of only di- and tetranucleotide repeats and sequences containing just one microsatellite, 767 sequences remained for further analysis. During primer selection, QDD grouped the primers into different primer classes (A to G). We chose only the two most restrictive classes, which fulfill the following conditions: (1) allowing assembled microsatellites, (2) not allowing nanosatellites in the primer region and (3) allowing nanosatellites in the flanking region, i. e., the region between the primer binding site and the actual microsatellite sequence (468 sequences). The final step was to select only sequences, whose microsatellites comprise a motif repeated eight to 22 times. After all this exclusions, 94 sequences retained for primer designing.

We selected primers with a high GC-content displaying no primer dimers and similar annealing temperature (comparing forward- and reverse primers of each primer pair). We tested the potential primer sequences against the original sequence with BioEdit version 7.2.5 [12]. In case we found no primers with matching annealing temperatures, we used NCBI Primer-BLAST [13] to find alternate primer sequences. We checked the primers for dimers with Amplify [14] and manually redesigned the primer sequences if necessary.

Finally 36 different microsatellite loci were left, which were first screened for consistent amplification via PCR. Those primers amplifying a product and working in different samples were chosen for microsatellite analyses of individuals from two surface populations (Wadi Al Falahi N = 24, Misfat Al Abriyeen N = 20). These individuals were collected in accordance with the local legislation of the Sultanate of Oman (Ministry of Environment and Climate Affairs: sampling permission number 13/2012, export permission number 29/2012).

Since we wanted to perform multiplex PCRs, we made use of the program Multiplex Manager version 1.0 [15], a tool for managing and optimizing a multiplex set by minimising the total number of reactions, minimising the annealing temperature differences among primer pairs within each reaction and maximising the size differences between the products amplified from the various loci within one reaction. The remaining 25 markers were put into three different multiplex reactions which were used for electrophoresis. For amplification these multiplex reactions were further divided as some loci showed a better readable electrophoresis pattern when amplified with a multiplex kit (QIAGEN Multiplex PCR Kit, QIAGEN, Netherlands) and others using the TopTaq kit (TopTaq DNA Polymerase, QIAGEN, Netherlands).

Multiplex PCR was performed either using the multiplex kit in 10 μl reaction volumes (1 μl DNA, 2.5 μl QIAGEN multiplex PCR mastermix, 1 μl primermix, 1 μl Q-solution and 4.5 μl AD), or using the TopTaq kit also with a reaction volume of 10 μl (0.5 μl DNA, 2 μl Q-solution, 1 μl buffer, 200 μM dNTPs, 1500 μM Mg++, 0.5 μl primermix, 0.5 units TopTaq-polymerase and 5.1 μl AD). According to the three different multiplex reactions, those PCRs were named MPR1/2/3 for multiplex kit reaction 1/2/3 or TTR1/2/3 for TopTaq kit reaction 1/2/3. We followed a two-step cycling protocol for each reaction as follows: initial denaturation at 95°C for 15 min, 2 cycles denaturation at 94°C for 30 s, annealing at 65°C (R1), 64°C (R2), 60°C (R3) for 90 s, extension at 72°C for 60 s. This was followed by 35 cycles denaturation at 94°C for 30 s, annealing at 60°C (R1), 59°C (R2), 54°C (R3) for 90 s, extension at 72°C for 60 s and a final extension at 60°C for 30 min. PCR products were diluted 1:20 with AD, multiplexed according to the reactions and added to a mixture of Hi-Di formamide and size standard (Gene Scan™ 500 LIZ, Life Technologies, USA). After a denaturation at 94°C for 4 min, the products were run on a 3130xl Sequence Analyzer (Applied Biosystems, USA).

After fragment analyses and testing for polymorphic loci, 19 different microsatellite markers proved to be suitable for genotyping. Primer sequences, PCR conditions, and their respective GenBank accession numbers are listed in Table 1. The alleles of all 19 loci (44 individuals) obtained from the resulting electropherograms were identified and binned with GENEMAPPER v5.0 (Applied Biosystems, USA) and manually reviewed. For each population, we estimated the observed (H_o_) and expected (H_e_) heterozygosities and tested for linkage disequilibria and deviations from Hardy-Weinberg equilibria using Genepop v 4.2.2 [16]. Subsequently we used Microchecker v 2.2 [17] to test for null alleles. We calculated the fixation Index with the program ARLEQUIN v3.5 [18]. All loci proved to be polymorphic in both populations. Results of population genetic analyses are shown in Table 2. For the 19 examined loci, the number of alleles per locus ranged from two to 20. Two loci (DL6X and 9XNC) showed a significant deviation on the 5% level from linkage equilibrium in both populations (Wadi Al Falahi and Misfat Al Abriyeen). Microchecker results revealed evidence for null alleles in one locus (CJHG; frequency after Oosterhout: 0.172) in the population Misfat Al Abriyeen and two loci (PH8A, 3ROZ; frequency after Oosterhout: 0.0815, 0.3868) in the population Wadi Al Falahi. However, since none of these loci showed a constant significant deviation in both populations, we did not exclude the loci from our analyses. Considering each population separately, one locus (CJHG) in the Misfat Al Abriyeen population and 4 loci (PH8A, JQSO, 88CM, 3ROZ) in the Wadi Al Falahi population were not in Hardy-Weinberg equilibrium displaying a deficit of heterozygotes, which is likely due to the occurrence of potential null alleles in the markers CJHG, PH8A and 3ROZ. None of the loci showed a significant deviation in the population Misfat Al Abriyeen as well as in the population Wadi Al Falahi. Heterozygosities at some loci were very low (UHPE) or even zero (3Z71, QLIM, 3 N43) for one of the populations. For the loci 3Z71, QLIM, 3 N43 this is due to the fact that the numbers of alleles are small (2–3) and in each case one of the two populations possessed only one of the alleles. In the locus UHPE the number of alleles is higher (7), but in each of the two populations one allele occurs with high frequency and most individuals are thus homozygous. The fixation index of 12.83% shows high degree of variation between the two populations (see Table 2 for single loci F_ST_ values).

**Table 1:** **Primer sequences**, **repeat motifs**, **amplification parameters and accession numbers of microsatellite loci in** ***Garra barreimiae***

Locus name	Primer sequences 5′ - > 3′	Primer labelling	Primer C (pMol)	Repeat motif	Reaction	T_a_(°C)	Accession Nr.
QLIM	F-ACGGTTGATAGAGCCACTGAC		2	AG	MPR1	65/60	KM099076
	R-ACTGTCGAGCAATGCAACAC	PET	2				
7GHL	F-GTATTACCGTGGTGATGTGGC	PET	4	AGAT	MPR1	65/60	KM099077
	R-GCGGCGTAGAAATCTGACTG		4				
PH8A	F-ACACTTCCAAAACGGTCGC	VIC	2	AGAT	MPR1	65/60	KM099078
	R-CGGAGCAAACCCAGTGACTA		2				
UHPE	F-ACCACAGCCAGTCAGATATGC		2	AC	MPR1	65/60	KM099079
	R-GCTCAACAAGCTGAACTCCG	6-FAM	2				
JQSO	F-TTGGCTGGAGCGATGGCTG	6-FAM	2	AC	MPR1	65/60	KM099080
	R-AGGGCTACATCACAGACTCAC		2				
DL6X	F-GTGACACATTTGGATAATGAATCAGC	NED	2	AC	TTR1	65/60	KM099081
	R-AGCTGGCCTAGATACCTGAC		2				
FYP9	F-CGTCGACCAACGGACTAACG	NED	2	AC	TTR1	65/60	KM099082
	R-GACCGACATCAATCACGCGCT		2				
HOLN	F-ACTGCGCTCGTACCCTATG	6-FAM	2	AC	TTR1	65/60	KM099083
	R-CAGCAGCCGGTAAATAGCTG		2				
JMLR	F-CGATCAGATATGCAATGGCAG		2	AC	MPR2	64/59	KM099084
	R-GTGATCAGGCCTGTGTAAACG	NED	2				
CJHG	F-CTGACCTGATCTACGAAACCG	6-FAM	1.6	AC	MPR2	64/59	KM099085
	R-TGAGTTTGTGGAGTCCAGCG		1.6				
3Z71	F-CGAGGTTTGGTTCCTCTTCTC	VIC	1.6	AG	MPR2	64/59	KM099086
	R-CAGCCTGGCTAACTCTGAGC		1.6				
9XNC	F-GCCAGTCAGTAGCTGCAAC	PET	1.6	AC	TTR2	64/59	KM099087
	R-GTTGTGCTATTCTGTGTAAGACC		1.6				
3 N43	F-GTGCTGCTTTCCTTCTTCG	PET	2	AC	TTR2	64/59	KM099088
	R-ACCAGGAGCCGATCCATAAAC		2				
1EHE	F-GCAGATGTAGTCAATTGACATGC	6-FAM	1	AC	TTR2	64/59	KM099089
	R-GCGAGGGCTTGAACTGATAC		1				
88CM	F-CTGTCGTCCATGGCTGTACC	VIC	1	AG	TTR2	64/59	KM099090
	R-AGGACTGGCATGTTTGCATT		1				
I6G2	F-AGCATCTGAGCATTCTCCTG	VIC	3	AT	MPR3	60/54	KM099091
	R-CCAAACGGTACGTCTTATGG		3				
3ROZ	F-TGGTGGTTTATATGCAGTCTGA	VIC	4	AC	MPR3	60/54	KM099092
	R-ACCCAAGGATGTTGTGTCCG		4				
CB75	F-CCATGCATCTACCTACCCAC	PET	2	AGGT	MPR3	60/54	KM099093
	R-TGATCAAATAGATGGTGGCTG		2				
2PUM	F-CATGCCAATTCACTCATTCC	NED	1	AG	TTR3	60/54	KM099094
	R-ACGTCACGTGTTGTCTATCTTG		1				

Primer C (pMol) primer concentration in pMol, Ta (°C) annealing temperature, Primer labelling: DS-33 Dye set; accession numbers.

**Table 2:** **Population genetic parameters of microsatellite loci of** ***Garra barreimiae*** **and cross**-**species amplification results**

Locus name	N MF	N WF	Size (bp)	N_A_	H_e_MF	H_o_MF	Oosterhout MF	HW p-value MF	H_e_WF	H_o_WF	Oosterhout WF	HW p-value WF	F_ST_	CA
QLIM^C^	20	24	88 - 92	3	0.00	0.00	0	NV	19.42	20.83	−0.108	0	0.059	22
7GHL	20	24	166 - 250	19	89.10	90.00	−0.022	0.571	93.09	95.83	−0.027	0.777	0.025*	11
PH8A^C^	20	24	126 - 188	15	86.03	95.00	−0.076	0.634	91.58	75.00	0.082	0.995	0.020*	21
UHPE^C^	20	24	89 - 101	7	35.26	30.00	0.107	0.872	54.43	62.50	−0.087	0.437	0.071*	20
JQSO^C^	20	24	147 - 169	7	74.49	75.00	−0.021	0.092	75.89	62.50	0.077	0.966	0.160**	20
DL6X	20	24	112 - 126	8	60.00	65.00	−0.069	0.250	59.75	70.83	−0.120	0.273	0.156**	19
FYP9^C^	20	24	169 - 235	20	78.46	90.00	−0.096	0.477	95.04	87.50	0.031	0.793	0.068**	22
HOLN^C^	20	24	191 - 233	7	59.49	65.00	−0.067	0.128	39.89	41.67	−0.033	0.419	0.195**	22
JMLR^C^	20	24	93 - 99	4	57.44	65.00	−0.064	0.360	71.37	70.83	−0.007	0.818	0.120**	20
CJHG^C^	20	24	198 - 215	7	54.23	35.00	0.172	0.968	77.66	75.00	−0.002	0.588	0.194**	21
3Z71^C^	20	24	123 - 127	2	29.62	35.00	−0.194	0.000	0.00	0.00	0	NV	0.170*	22
9XNC^C^	20	24	101 - 112	6	71.67	70.00	−0.012	0.594	74.20	70.83	0.007	0.589	0.061*	22
3 N43	20	24	182 - 184	2	0.00	0.00	0	NV	28.37	33.33	−0.184	0	0.135*	22
1EHE^C^	20	24	106 - 112	4	68.08	80.00	−0.106	0.000	63.03	62.50	0.004	0.068	0.176**	22
88CM^C^	20	24	190 - 198	6	40.90	45.00	−0.069	0.000	73.40	54.17	0.120	0.995	0.216**	21
I6G2^C^	20	24	115 - 137	8	58.21	60.00	−0.012	0.684	71.45	62.50	0.048	0.781	0.282**	22
3ROZ	20	24	262 - 276	5	14.23	5.00	0.172	0.923	56.34	4.55	0.386	1.000	0.172*	0
CB75	20	24	171 - 199	7	51.41	45.00	0.067	0.767	68.17	75.00	−0.069	0.074	0.005	2
2PUM^C^	20	24	152 - 176	8	38.33	45.00	−0.240	0.000	77.66	87.50	−0.082	0.212	0.121**	22

N number of individuals analysed, Ta (°C) annealing temperature, NA number of alleles, MF Misfat Al Abriyeen, WF Wadi Al Falahi, H_e_ expected heterozygosity in%, H_o_ observed heterozygosity in%, null allele estimates (Oosterhout values), HW p-value probability for deviation from Hardy-Weinberg equilibrium, F_ST_ values (*indicates significant values at p < 0.05 and **at p < 0.001); loci marked with ^C^were positive and polymorphic in *Garra rufa*; CA number of individuals positive in cross-species amplification (tested in 22 individuals of *G. rufa*).

All markers were tested for cross-species amplification in 22 individuals of *G. rufa* (fin clips were taken from bred individuals kept in the Zoo Vienna). Out of the 19 loci, 14 were positive in *G. rufa* and proved to be polymorphic in at least 75% of the individuals (Table 2). Thus, these 14 loci could be used for population genetic analyses in the commercially exploited species *G. rufa*.

The set of microsatellite loci isolated and tested in the present study will be useful DNA markers for further population genetic studies of *G. barreimiae* and might also be used in other species of this genus. The results from using these microsatellite markers will provide deeper insights into the species’ population structure and its genetic diversity. Furthermore, it will be of utmost interest to use these loci in the cave population of *G. barreimiae* and estimate gene flow between the cave and the surface population. This will help to evaluate species delimitation of *G. barreimiae* and to assess whether any cryptic species exist besides *G. barreimiae*.
